# 822. Managing Burn Autograft Donor Site Pain Using Antimicrobial Bioresorbable Lidocaine-HCL Matrix: A Five-Patient Case Series

**DOI:** 10.1093/jbcr/irag033.261

**Published:** 2026-04-06

**Authors:** Siva Paladugu, Jonathan E Schoen, Jeffrey E Carter, M Victoria P Miles

**Affiliations:** Burn Center at University Medical Center, New Orleans, LA; UCI Health Regional Burn Center, New Orleans, LA; University Medical Center Burn Center, Louisiana State University Health Sciences Center New Orleans, New Orleans, LA; University Medical Center Burn Center, Louisiana State University Health Sciences Center, Department of Surgery, New Orleans, New Orleans, LA

## Abstract

**Introduction:**

Autograft donor sites in burn patients are associated with significant post-procedural pain and infection risk, contributing to delayed recovery, increased hospital costs, and reliance on systemic analgesics. A bioresorbable polymer matrix incorporating lidocaine HCl with metallic and ionic silver was developed to address these challenges. Upon application, the antimicrobial matrix conforms to the wound bed, facilitates a moist healing environment, and releases lidocaine in a dose-controlled manner. This case series evaluated feasibility, patient pain scores, and preliminary efficacy of this matrix in donor site management.

**Methods:**

Patients undergoing donor site harvesting at a single ABA-verified burn center from July–September 2025 received application of the matrix in 4x6cm sheets per manufacturer’s instructions. Factors related to ease of matrix use and application were graded on a 5-point Likert scale, and pain scores on a 10-point scale were collected at baseline, 45 minutes, and 3 hours post-operatively. Data on application, infection, systemic analgesic needs, and wound management were recorded. Per institutional policy, this study was exempt from IRB oversight.

**Results:**

Five patients (4 adults, 1 pediatric) were treated with one to five sheets depending on donor wound size. Product usability was rated as good to excellent across all cases, with factors such as application, adherence, and conformability each consistently receiving a score of 3-5 out of 5. Additional wound management techniques included skin cell suspension autograft and secondary dressings. Systemic pain management varied between the patients. Average reported pain scores were 0 at baseline, 2.0 at 45 minutes, and 2.6 at three hours post-operatively. No cases of infection were documented, and the matrix was considered well-suited by treating physicians.

**Conclusions:**

This five-patient case series demonstrates that the bioresorbable antimicrobial matrix with lidocaine HCl is a feasible adjunct for burn donor site management. The matrix was easy to handle, conformable, and deemed appropriate for donor site wounds. Pain scores were generally mild supporting its potential to limit opioid use. The absence of infections further supports its antimicrobial efficacy. Larger studies are needed to confirm benefits and optimize use.

**Applicability of Research to Practice:**

The straightforward application and conformability of the matrix offer a practical approach for burn autograft donor site management. By simplifying wound care and lessening opioid dependence, this matrix may help to support faster recovery and enhance overall patient care.

**Funding for the study:**

N/A.

